# A Map of Consciousness Studies: Questions and Approaches

**DOI:** 10.3389/fpsyg.2020.530152

**Published:** 2020-10-08

**Authors:** Takuya Niikawa

**Affiliations:** ^1^Institut Jean Nicod, ENS, Paris, France; ^2^Faculty of Humanities and Human Sciences, Hokkaido University, Sapporo, Japan

**Keywords:** consciousness, consciousness science, philosophy of consciousness, approach to consciousness, questions about consciousness

## Abstract

This article aims to present a map of consciousness studies, which consists of a list of fundamental questions about consciousness and existing approaches to them. The question list includes five fundamental categories: Definitional, Phenomenological, Epistemological, Ontological, and Axiological. Each fundamental category is divided into more determinate questions. Existing approaches to each question are also classified into a few groups, presenting principal researchers who take each kind of approach. In the final section, I demonstrate the usefulness of the proposed map of consciousness studies by applying it to examine the integrated information theory and the global workspace theory of consciousness.

## Introduction

Academic research starts with research questions. An area of research typically develops by research questions being sophisticated, in particular, those being conceptually clarified and being divided into more determinate questions. In the philosophy of language, for instance, the research question of “what is the meaning of symbols?” was divided into two different types of questions, namely, the question about semantics—“what is the meaning of this or that symbol (for a particular person or group)?”—and the question about foundation—“in virtue of what facts about that person or group does the symbol have that meaning?” (Speaks, 2019, sec. 1). This division has helped us to develop theories of meaning without confusion. In linguistics, likewise, the research question of “what is the linguistic capacity?” can be divided into two distinct questions, namely, the competence question—“what is the linguistic competence?”—and the performance question—“what is the linguistic performance ability?” (Chomsky, 2014). This distinction helps us to develop theories of linguistic capacities while avoiding unnecessary confusion.

Consciousness studies have rapidly developed in the last three decades; many philosophical and scientific theories of consciousness have been proposed. However, it is far less clear how such theories of consciousness are related to each other. Some theories target different aspects of consciousness; some theories address the same aspect of consciousness but with different methodologies. Consider two influential scientific theories of consciousness, the integrated information theory (IIT) (Tononi, 2008; Tononi et al., 2016) and the global workspace theory (GWT) (Baars, 2005; Dehaene, 2014). Although many assume that they are competitive, it is unclear whether they are concerned with the same research subject in the first place (Ball, 2019). Given that there already exist many theories of consciousness, and it is far less clear how they are related, we need to stop trying to answer a specific research question set out in a theoretical framework for a moment and instead *take research questions about consciousness themselves as the target of investigation*. In other words, a *second-order investigation* of the research questions about consciousness is required to further develop consciousness studies.

As an initial step of the second-order investigation, this article presents *a systematic list of questions about consciousness* (see section “The List of Questions”). This list helps us to understand what questions the existing theories of consciousness address. In addition, the list helps each consciousness researcher to see what aspects of consciousness they are interested in.

After proposing a list of questions about consciousness, I also submit a list of *approaches* to each question (see section “The List of Approaches”). The list of approaches gives us the methodological overview of consciousness studies. It also helps researchers working in various fields to see what question they can tackle in their methodological/theoretical frameworks.

The list of questions is constructed by a *top–down approach*. I apply the traditional taxonomy of philosophical inquiries to categorize questions about consciousness. Thus, the proposed classificatory framework is neither arbitrary nor groundless. The list of approaches is constructed by a *bottom–up approach*. I take the existing approaches to each kind of question and then classify them based on their crucial methodological differences.

The final section of this article is dedicated to demonstrate the usefulness of the map of consciousness studies, which consists of the lists of questions and approaches, by applying it to examine IIT and GWT. I will argue that the proposed map is useful in that it can provide a multidimensional framework in which to compare various scientific theories of consciousness, including IIT and GWT.

## The List of Questions

Philosophical inquiries have typically been divided into three categories: *Ontological*, *Epistemological*, and *Axiological* (Lee, 1966, p. 72; Woleński, 2004, p. 3). In addition to this traditional distinction, I incorporate two other fundamental categories into the classificatory framework for questions about consciousness, namely, *Definitional* and *Phenomenological*. Definitional inquiries explore satisfactory definitions of key concepts, such as “good” and “knowledge.” The term “consciousness” is also a target of this inquiry. Phenomenology is a discipline in which to investigate conscious phenomena from the subjective point of view, which is typically distinguished from other disciplines of philosophy (Smith, 2018, sec. 1). There is no doubt that the category of phenomenology should be included in the classificatory framework for questions about consciousness.

**FIGURE 1:**
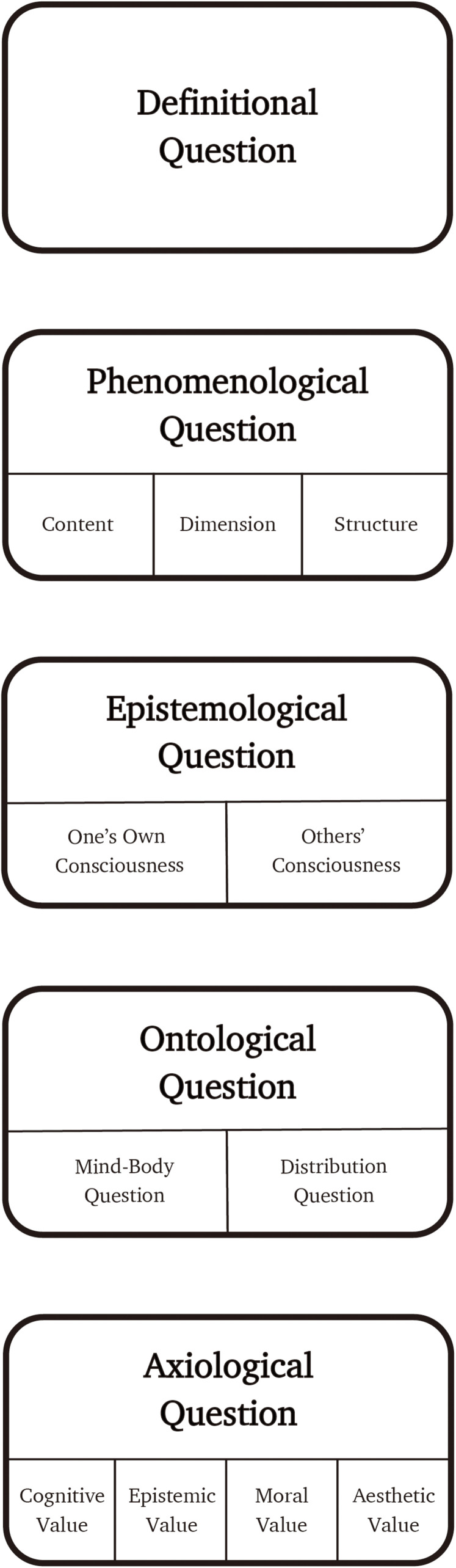
The list of questions about consciousness.

Thus, we have five fundamental categories in which questions about consciousness are classified: *Definitional*, *Phenomenological*, *Epistemological*, *Ontological*, and *Axiological*^1^. Each fundamental category (except the definitional) has subcategories. The subcategories are set out partially in a bottom*–*up manner: it is partially based on the widely accepted division in the subject matter. In the rest of this section, I present the five fundamental questions about consciousness and how they are divided into subquestions (Figure 1).

### Definitional Question

How should we define the term “consciousness” and its cognates?

The definitional question is not divided into subquestions….

### Phenomenological Question

What phenomenological features does consciousness have?

The phenomenological question is divided into three subquestions, depending on what aspect of consciousness to focus on, namely, *content*, *dimension*, or *structure*. The content of consciousness is understood as *variable* features of consciousness, such as experienced color, shape, movement, taste, or feel^2^. The dimension of consciousness is understood as the fundamentally different kinds of conscious experiences, such as perceptual, cognitive, and emotional dimensions (Kriegel, 2015). The structure of consciousness is understood as *invariable* features of consciousness, such as unity and figure-ground structure (Bayne, 2010; Watzl, 2011; Macpherson, 2015). While the general structures of consciousness itself are typically discussed in the philosophy of consciousness, the specific structures of each dimension of consciousness can also be investigated.

#### Content Question

What content does consciousness have?

#### Dimension Question

What dimensions does consciousness have?

#### Structure Question

What structures does consciousness have?

### Epistemological Question

How do we know about consciousness?

The epistemological question is divided into two subquestions, depending on whose consciousness to address, whether *one’s own consciousness* or *the consciousness of others*.

#### Epistemological Question About One’s Own Consciousness

How do we know about our own consciousness?

#### Epistemological Question About Others’ Consciousness

How do we know about the consciousness of others?

### Ontological Question

How is consciousness located in the world?

The ontological question is divided into two subquestions^3^. The first concerns the relation between consciousness and the physical world; the second concerns the distribution of consciousness over the physical world.

#### Mind*–*Body Question

What relation holds between consciousness and the physical world (in particular our brain)?

#### Distribution Question

How is consciousness distributed in the physical world? (In other words, what has consciousness?)

### Axiological Question

What values does consciousness have?

This question is divided into four subquestions, depending on what kind of value to address, namely, *cognitive*, *epistemic*, *moral*, or *aesthetic*^4^.

#### Cognitive Value Question

What type of cognitive capacity does consciousness enable its possessor to have?

#### Epistemic Value Question

What type of knowledge does consciousness enable its possessor to have?

#### Moral Value Question

What type of moral status does consciousness enable its possessor to have?

#### Aesthetic Value Question

What type of aesthetic value does consciousness enable its possessor to have?

## The List of Approaches

**FIGURE 2:**
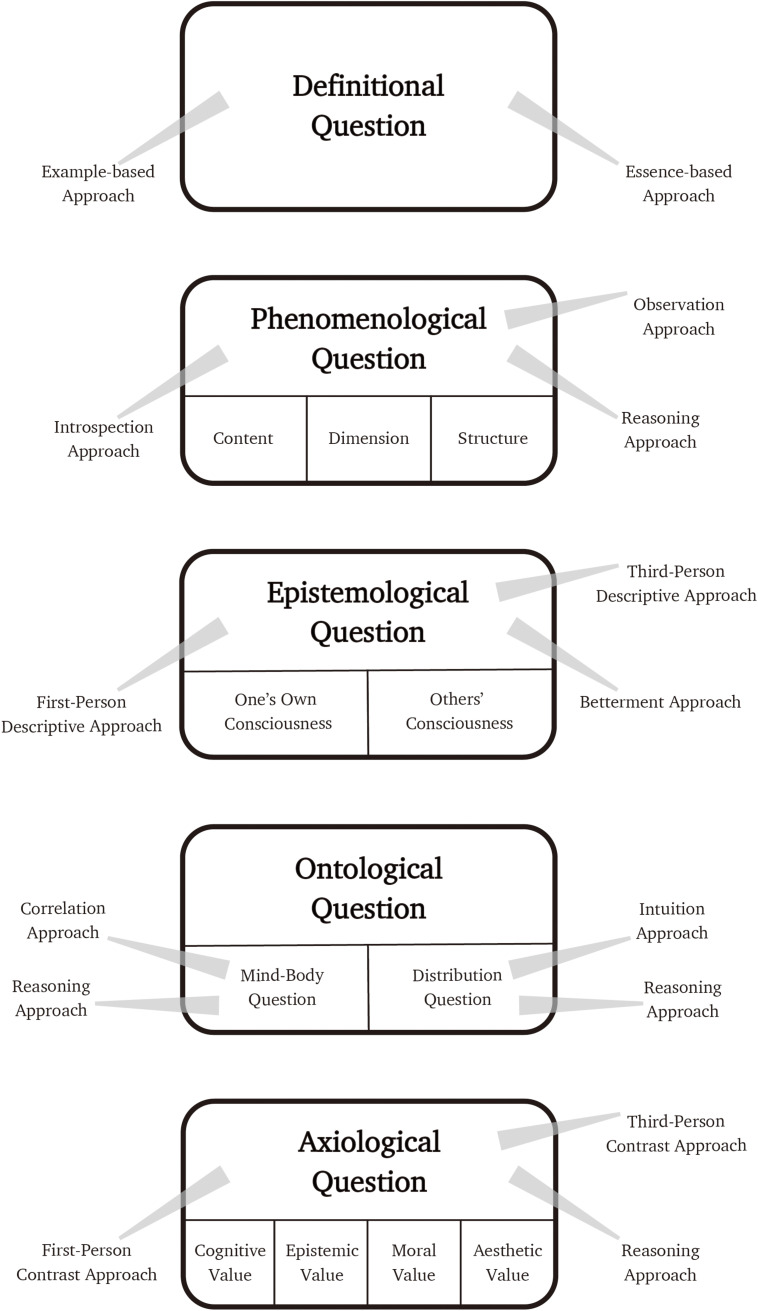
The list of questions about consciousness and each approach to them.

In this section, I present approaches to each kind of questions that have been actually employed by consciousness researchers with a brief assessment of them (Figure 2). Note that although each approach can be taken individually to address one question, we can also take different approaches in combination to address one specific question. In this sense, these approaches are not exclusive.

### Approaches to the Definitional Question

Let us start with the definitional question: How should we define the term “consciousness” and its cognates? There are two approaches to the definitional question: (I) *example-based approach* and (II) *essence-based approach.*

The example-based approach defines the term “consciousness” as *something* that is shared by typical examples of conscious states/experiences, such as pain experience and visual experience (Velmans, 2009; Nida-Rümelin, 2016; Prinz, 2016; Schwitzgebel, 2016). This approach can provide a theoretically neutral definition of consciousness, since it does not refer to any distinctive property in the definition of the term “consciousness.” The problem of the example-based approach is that it is unclear how we should determine the scope of typical examples of conscious experiences. If we restrict the “typical” examples of conscious experiences too narrowly, borderline cases of consciousness, such as dreamless sleep and vegetative states, may be automatically excluded from consciousness studies. However, it is controversial whether dreamless sleep and vegetative states are conscious states (Shea and Bayne, 2010; Windt et al., 2016).

The essence-based approach defines the term “consciousness” by referring to its essential property such as phenomenality (or “what-it-is-like-ness”) (Chalmers, 1997), the property of being inner, qualitative, and subjective (Searle, 2000), and being accurately reportable (Baars, 1993, p. 19). The merit of this approach is that it can provide an informative definition of the term “consciousness.” Its problem is that it is highly controversial what property we should count as the definitional property of consciousness; there may be no single property that all the consciousness researchers accept to be essential for consciousness. Thus, the essence-based approach may cause a dispute over the correct definition of consciousness. This dispute can be resolved if we take a pluralist position about the definition of consciousness, allowing that there are several different notions of consciousness defined in different manners, such as “phenomenal consciousness” and “access consciousness” (Block, 1995). However, meta-level questions arise for the pluralists: what relation holds between those notions of consciousness? Are they different aspects of a single phenomenon, or do they refer to different phenomena?

### Approaches to the Phenomenological Question

The second question is the phenomenological question: What phenomenological features does consciousness have? This question is divided into three subquestions: content, dimension, and structure. There are three approaches to each subquestion: (I) *introspection approach*, (II) *observation approach*, and (III) *reasoning approach*.

The introspection approach explores the phenomenological features of conscious experiences by *introspection in a broad sense*, where it involves not only the cognitive activities of “turning one’s attention inward and attending to one’s own concurrent internal goings-on” but also uses of memory, imagination, and concept application (Kriegel, 2015, pp. 20, 21). Thus, introspection in this sense can include imagining a conscious experience and conceptually describing its phenomenological feature; it can also involve imaginatively comparing a current conscious experience with past conscious experiences^5^.

The introspection approach works for basic contents of conscious experiences and their salient dimensions and structures. For instance, when I introspect on my current conscious experience of drinking Springbank 15 years, I can find that it has sweetness, smokiness, saltiness, and its distinctive sulfur smell as its flavor contents, that it has temporal continuity as its structure, and that it has perceptual and algedonic dimensions. However, there are cases for which the introspection approach does not work. For instance, sophisticated skills of introspection are required to capture the complex phenomenological features of consciousness such as dynamical interactions of attentional shifts and flavor profiles of drinking Springbank 15 years, but people typically lack such skills. Furthermore, introspection seems unable to determine whether a perceptual experience can have causal relations (e.g., touching a screen causing the screen flashing) and natural kind properties (e.g., being water) as its content (Siegel, 2007) and whether consciousness has cognitive phenomenology as a distinct dimension (Spener, 2011). This is not because our skills of introspection are not sophisticated to the required extent, but because it is unclear how introspective data are related to these issues. We need to clarify what introspective data are predicted if we consciously experience causal relations between events in addition to experiencing sequential occurrences of events, if we consciously experience the property of being water in addition to experiencing the presence of clear, colorless liquid, and if consciousness has a cognitive dimension as being irreducible to other dimensions such as sensory and imaginative ones. Introspection turns out useless if no introspectable difference is predicted there. A more fundamental limitation of introspection is that one’s introspection is not effective to understand the phenomenological features of conscious experiences that one is *unable to have*. For instance, it is hard for normal people to understand by introspection the phenomenological features of schizophrenic experiences and synesthetic experiences.

The observation approach is to infer what phenomenological features a conscious experience has from its possessor’s observable states/behaviors, including their subjective reports. For instance, when one reports that she sees a red patch, then we can infer that her conscious experience has red-color content; when one groans painfully, we can infer that she has pain experience^6^. This approach is available to explore the phenomenological features of conscious experiences that are difficult for researchers to have by themselves, such as schizophrenic and synesthetic experiences. We can, in principle, infer what phenomenological features such conscious experiences have from their possessors’ observable states/behaviors, in particular, their introspective reports (Hubbard and Ramachandran, 2003; Fuchs, 2007; Simmonds-Moore, 2016). Experimental neuroscience of consciousness typically employs the observation approach in investigating neural properties responsible for the phenomenological features of conscious experiences (Tononi and Koch, 2015, sec. 3). This is in part because it needs to collect quantitative data about neural activities from *many experimental participants* rather than the researchers themselves alone. The practical problem with this approach lies in how to interpret the observable states/behaviors. In addressing untypical conscious experiences, even the introspective reports of such experiences are difficult to interpret. Here is a report from a patient with depersonalization syndrome: “I feel as though I’m not alive as though my body is an empty, lifeless shell. I seem to be standing apart from the rest of the world, as though I’m not really here” (Bockner, 1949, p. 969). It is hard to understand what the patient’s experience is like. Furthermore, Michel (2019) points to the crucial role of background beliefs in interpreting observable states/behaviors, claiming that the disagreements among consciousness scientists mainly lie in whether to interpret certain observable states/behaviors as evidence for the presence of a phenomenological feature.

The *reasoning approach* infers what phenomenological features consciousness has from a given thesis about consciousness. For instance, Pitt (2004) argues that given that the possession of consciousness enables us to introspectively know what we think, consciousness must have a cognitive dimension as being irreducible to other dimensions^7^. A challenge to those who adopt this approach is to justify a thesis about consciousness used as a premise for reasoning. Note that the premise in Pitt’s reasoning can be counted as an answer to the epistemic value question: What type of knowledge does consciousness enable its possessor to have? As shown in this, answers to other kinds of questions, in particular, the *ontological question* and the *axiological question*, can possibly be the premises used in the reasoning approach.

### Approaches to the Epistemological Questions

The third question is the epistemological question: How do we know about consciousness? This question is divided into two subquestions depending on whose consciousness to address, namely, one’s own consciousness or the consciousness of others. There are three approaches to the epistemological question: (I) *first-person descriptive approach*, (II) *third-person descriptive approach*, and (III) *betterment approach*.

The first-person descriptive approach describes how we know about our own consciousness and the consciousness of others from the first-person perspective. To describe how one knows about one’s own consciousness from the first-person perspective is to describe first-personally *the processes of introspection*. This approach is employed in constructing/assessing theories of introspection. For example, some philosophers reflect on the process of introspection on perceptual experience and describe it as being “transparent” in that we know the contents of our own perceptual experience through being aware of the external objects/events (Harman, 1990; Tye, 2000). A theory of introspection is constructed/assessed partially based on the transparency of perceptual experience. If a theory of introspection implies that introspection is entirely distinct from perceptual awareness, the theory seems to conflict with the transparency of perceptual experience and therefore be assessed negatively. Likewise, to describe how one knows about the consciousness of others from the first-person perspective is to describe first-personally *the processes of knowing others’ conscious experiences*. Some philosophical and phenomenological accounts of how to know others’ conscious experiences are partially based on the first-personal descriptions of such processes (Wittgenstein, 1980, sec. 570; Scheler, 2008; Overgaard, 2017).

The third-person descriptive approach describes how we know about our own consciousness and the consciousness of others from the third-person perspective. To describe how one knows about one’s own consciousness from the third-personal perspective is to describe third-personally the processes of introspection. This approach typically focuses on the neural/psychological processes responsible for introspection (Fleming et al., 2010; Baird et al., 2013; Jacobs and Silvanto, 2015), where introspection is not differentiated from metacognition [for the conceptual relation between introspection and metacognition, see Overgaard and Sandberg (2012, sec. 1)]. A theory of introspection can also be evaluated based on the relevant scientific findings. Likewise, to describe how one knows about the consciousness of others from the third-personal perspective is to describe third-personally the processes of knowing others’ conscious experiences. This approach includes attempts to describe the processes of *mind reading* and *empathy*. Some focus on the relevant neural/psychological processes (Marsh, 2018), others address external conditions in which we try to know others’ conscious experiences (Gallagher and Hutto, 2008). A theory of mind reading/empathy can be constructed/assessed based on the data acquired through this type of exploration.

The betterment approach explores how we can better know about consciousness, rather than just describing how we know about it. There are a few research projects that can be counted as the betterment approach. Some training programs to enhance the skills of empathy have been developed (Lam et al., 2011; Englander, 2014). Likewise, there is a research project to design a training program to enhance the skills of introspection in general (Miyahara et al., 2020). There are also many attempts to invent an interview-based method to know better what others consciously experience (Petitmengin, 2006; Langdridge, 2007; Giorgi, 2009; Petitmengin et al., 2019). Moreover, brain-decoding techniques may be available to know better about the consciousness of others, including behaviorally non-responsive patients’ experiences (Naci et al., 2017). Importantly, we can see the betterment approach as developing methods to address the phenomenological question^8^.

### Approaches to the Ontological Question

The fourth question is the ontological question: How is consciousness located in the world? This question is divided into two subquestions. The first is the mind–body question: What relation holds between consciousness and the physical world (in particular our brain?) The second is the distribution question: How is consciousness distributed in the physical world? (In other words, what has consciousness?) There are two approaches to the mind–body question: (I) *correlation approach* and (II) *reasoning approach*. There are also two approaches to the distribution question: (I) *intuition approach* and (II) *reasoning approach*.

The correlation approach explores what neural or informational feature is correlated with the presence of a phenomenological feature of consciousness (or the presence of consciousness itself) by using brain scanning technologies such as functional magnetic resonance imaging (fMRI) and brain stimulation techniques such as repetitive transcranial magnetic stimulation (rTMS). Simply put, this is to explore “neural correlates of consciousness (NCC),” which are the minimal neuronal mechanisms jointly sufficient for a specific content, dimension, or structure of consciousness (or the presence of consciousness itself) (Crick and Koch, 1990). There are many findings of the neural and informational correlates of consciousness (Koch et al., 2016; Boly et al., 2017; Wu, 2018, secs 4, 5). For example, some found that the conscious experience of a visual scene is correlated with the activities of the parahippocampal place area of our brain (a subregion of the parahippocampal cortex that lies medially in the inferior temporo-occipital cortex) (Mégevand et al., 2014); others found that the conscious experience of a human face is correlated with the activities of the posterior and mid fusiform gyrus (Parvizi et al., 2012). The limitation of the correlation approach is that it cannot, in principle, reveal a more substantial relationship between consciousness and the physical world than the correlation relation. Since the correlation relation is consistent with many metaphysical relations such as causal relation, grounding relation, and identity relation, the correlation approach cannot determine which metaphysical relation holds between consciousness and the physical world (Kozuch and Kriegel, 2015).

The reasoning approach infers what relation holds between consciousness and the physical world from a given thesis about consciousness. For example, Papineau (2002, pp. 31–35) takes the causal efficacy thesis that consciousness can cause physical effects as a key premise for reasoning and argues that consciousness is identical to physical properties. Chalmers (2010, pp. 106–108) takes the conceivability of a phenomenal zombie—the thesis that it is conceivable that a physical duplicate of us lacks consciousness—as a key premise for reasoning and argues that consciousness cannot be physical. Campbell (2002, chap. 6) argues that perceptual consciousness must be constituted by ordinary mind-independent objects on the premise that perceptual consciousness enables its possessor to know about such ordinary mind-independent objects demonstratively. As shown in these examples, the reasoning approach can address what metaphysical relation holds between consciousness and the physical world beyond mere correlation. As we saw in section “Approaches to the Phenomenological Question”, however, a challenge to those who adopt this approach is to justify the thesis about consciousness used as a key premise for reasoning. To address this, for example, one may try to justify the causal efficacy thesis by appealing to our folk psychological briefs, such as the one that “my conscious thirst caused me to fetch a beer” (Papineau, 2002, p. 21); another may try to justify the conceivability of a phenomenal zombie by providing an argument against the *a priori* entailment from physical facts to phenomenal facts. The essential difficulty with the reasoning approach is to settle the conflicts between those who take distinct theses, which are justified in different manners, as the premises to infer opposing ontological positions (such as physicalism and anti-physicalism).

The intuition approach to the distribution question asks our intuition what has consciousness. We typically have some intuitive thoughts about what can have consciousness. For instance, it seems doubtless to me that other human beings are conscious. Many other kinds of mammals, such as dogs and cats, seem to have consciousness. However, microphysical entities and machines like my laptop do not seem to have consciousness. It is unclear to me whether insects and plants are conscious. On the assumption that intuition is a reliable epistemic route to know about the distribution of consciousness over the world, we can employ our intuition to answer the distribution question. The obvious problem with this approach is to justify the assumption that intuition is reliable with respect to the distribution of consciousness over the world.

The reasoning approach is also available to address the distribution question: to infer what has consciousness from a given thesis about consciousness. For example, if we take biological naturalism that consciousness is a biological phenomenon (Searle, 1992) as a premise for reasoning, we can infer that non-biological entities, such as machines and robots, cannot have consciousness. If we take IIT that consciousness is identical to internally generated and integrated information (Tononi, 2008) as a premise for reasoning, we can conclude that any system that generates information in an integrated manner has consciousness (for the detail of IIT, see section “Applications: Integrated Information Theory and Global Workspace Theory”). As we have seen, the essential difficulty with this approach is to settle the debates between those who take distinct theses, which are justified in different manners, as the premises for reasoning.

Note that the answer to the distribution question directly affects the scope of the phenomenological and epistemological questions. For instance, since IIT implies that computers which generate information in an integrated manner possess consciousness, the question of how we can know about the consciousness of such computers arises for advocates of IIT. Likewise, IIT opens up the phenomenological question about such computers: What content, dimension, and structure does their consciousness have?

### Approaches to the Axiological Question

The fifth question is the axiological question: What values does consciousness have? This question is divided into four subquestions depending on what kind of value to address: cognitive, epistemic, moral, and esthetic. The scope of those subquestions is not restricted to the values of *consciousness itself* but includes those of each content, dimension, and structure of consciousness. There are three approaches to the axiological question: (I) *first-person contrast approach, (II) third-person contrast approach*, and (III) *reasoning approach*.

The first-person contrast approach explores what difference there is in relevant value between the cases where one has and lacks consciousness (or where one’s consciousness has and lacks a specific phenomenological feature) from *the first-person perspective*. This approach typically consists of the following two steps: (a) to first-personally imagine that one loses consciousness (or a specific phenomenological feature disappears from one’s consciousness) and (b) to consider what value-related feature she thereby loses. Siewert (1998, 2014) takes this approach, arguing that (1) consciousness makes the life of its possessor worth living and that (2) only the possessor of consciousness can perform intentional cognitive activities/processes such as making judgments and having desires. The first point concerns moral value and possibly aesthetic value; the second point concerns cognitive value. Campbell (2002, chap. 1) also takes this approach, arguing that perceptual experience enables its possessor to know about ordinary mind-independent objects demonstratively. The problem with this approach is that it is controversial whether our first-personal thoughts about the values of consciousness are reliable. When reflecting on how we visually discriminate an object from others, for example, we are likely to think that if we lose perceptual consciousness, we cannot carry out the discrimination task. However, this intuitive thought seems to be falsified by the case of blindsight: the patient can achieve various visual discrimination tasks by “guesswork,” even though he said he did not have any visual experience (Weiskrantz, 2007).

The third-person contrast approach explores what difference there is in relevant value between the cases where one has and lacks consciousness (or where one’s consciousness has and lacks a specific phenomenological feature) from *the third-person perspective*. Dehaene and Naccache (2001) take this approach, arguing that consciousness enables durable and explicit information maintenance, novel combinations of operations, and intentional behavior. Weiskrantz (1997, chap. 7) focuses on a broad spectrum of syndromes in which there seems to be a loss of capacities related to consciousness, such as blindsight and aphasic disorders, arguing that consciousness grounds the capacities to perform flexible thinking and imagining. Kriegel (2017) compares our natural attitudes to conscious beings and non-conscious beings, and argues that consciousness confers dignity as a moral status on its possessors (possibly with some other conditions). However, it may be objected that the apparent differences in values can be explained without appealing to consciousness (Lau, 2009; see also Rosenthal, 2008). Hence, a challenge to the third-person contrast approach is to argue that the proposed difference in a type of value cannot be well explained without referring to consciousness.

The reasoning approach infers what values consciousness has from a given thesis about consciousness. For example, Tye (1996) takes the representationalist thesis that consciousness is representational as the key premise for reasoning and concludes that consciousness enables its possessor “to do a wide variety of things that they would not be able to do without it—for example, to recognize objects, to avoid knocking into them” (pp. 301, 302). The proponents of the attentional schema thesis that consciousness is an internal model of attention (Graziano and Webb, 2014) can take it as a premise for reasoning and conclude that consciousness enables its possessor to control attention in proper manners.

## Applications: Integrated Information Theory and Global Workspace Theory

This article proposes a map of consciousness studies, which consists of a systematic list of questions about consciousness and existing approaches to each question. In this final section, I apply this map to examine IIT and GWT. I first address how IIT answers each fundamental question that I have listed. In doing so, I point out several challenges to IIT. I then take the same procedure to examine GWT. I finally propose a way to clarify the relation between IIT and GWT with the help of the proposed map of consciousness studies. The discussion is sketchy but still sufficient to demonstrate how the proposed map can be used to examine and compare theories of consciousness.

Let us start with the definitional question. Tononi (2015, abstract, emphasis added) claims that IIT “attempts to identify the *essential properties* of consciousness (*axioms*) and, from there, infers the properties of physical systems that can account for it (postulates).” He lists five essential properties of consciousness, namely, intrinsic existence, composition, information, integration, and exclusion, and calls them “axioms” (Tononi, 2015, sec. 2). The intrinsic existence axiom states that consciousness exists independently from external observers, the composition axiom states that consciousness is structured, the information axiom states that each conscious experience is the particular way it is and thereby it differs from other possible conscious experiences, the integration axiom states that consciousness is unified, and the exclusion axiom states that consciousness is definite in content and spatiotemporal grain^9^. The fact that they are called “axioms” suggests that the conjunction of the listed essential properties fixes the *reference* of “consciousness.” Thus, IIT takes the essence-based approach to the definitional question, claiming that consciousness is defined in terms of the five axioms.

One slogan of IIT is that it goes “from phenomenology to physics” (Tononi et al., 2016, p. 450); the axioms are called the “phenomenological axioms” (Oizumi et al., 2014). This indicates that the axioms are derived from phenomenological considerations, namely, by addressing the phenomenological question, in particular, the structure question of what invariant features consciousness has (since the essential properties of consciousness are the invariant of consciousness). This suggests that advocates of IIT answer the definitional question through tackling the structure question.

Advocates of IIT claim that the phenomenological axioms “cannot be doubted and do not need proof” and are “taken to be immediately evident” (Oizumi et al., 2014, p. 2). This shows that they take the introspection approach to the structure question, rather than the observation approach and the reasoning approach, to derive the phenomenological axioms. However, some philosophers cast doubt on the plausibility of the axioms as capturing the essential phenomenological features of consciousness (Bayne, 2018; Pokropski, 2018; Miyahara and Witkowski, 2019). This demonstrates that the phenomenological axioms *can be doubted* and should not be taken to be immediately evident. Thus, advocates of IIT must justify the phenomenological axioms, employing the other approaches if needed.

Let us next move onto the ontological question. IIT specifies five informational features of physical systems (so-called “postulates”), each of which is supposed to account for a corresponding phenomenological axiom, and states that every physical system that realizes the five postulates possesses consciousness^10^. This statement is counted as the answer to the distribution question. Nevertheless, it is not fully clear what reasoning is in play here (especially in what sense each postulate “accounts for” a corresponding phenomenological axiom and why each postulate necessitates the phenomenological feature represented by the corresponding axiom). In order to evaluate IIT’s answer to the distribution question, thus, we should clarify the exact premises and inferential steps that constitute the reasoning in question.

IIT answers the mind–body question by stating that conscious experience is identical to an integrated informational structure of physical systems that instantiates the five postulates (Tononi, 2015, sec. 4). There is, however, no mention of how the identity claim is derived in any IIT literature. As we have seen in section “Approaches to the Ontological Question”, identity is not reasonably inferred only from the presence of correlation, since other metaphysical relations such as causal relation and grounding relation are also compatible with the presence of correlation. To justify the identity claim, advocates of IIT need to clarify what theses they use as the premises for the reasoning in question, in addition to the experimental finding that there is a correlation between the presence of consciousness and a relevant informational structure of brains (Massimini et al., 2005). Otherwise, we cannot properly evaluate IIT’s identity claim.

Let us finally examine what implications IIT have for the epistemological and the axiological questions. First, IIT seems to have an implication for the epistemological question about the consciousness of others. IIT states that the phenomenological features of consciousness (in particular contents and dimensions) are reflected in the *form* of the informational structure of physical systems (Tononi, 2015, sec. 4; Tononi et al., 2016, p. 459). It follows from this that we can infer the phenomenological features of the consciousness of others from the form of the informational structure of their brain, which we can, in principle, specify from the third-person perspective. This can be counted as an answer to the epistemological question about the consciousness of others. IIT also has an implication for the cognitive value question. If it is cognitively advantageous for physical systems to generate information in an integrated manner, IIT implies that the possession of consciousness is cognitively advantageous for that very reason.

I turn to how GWT (in particular its major advocate Stanislas Dehaene) answers each fundamental question listed in The List of Questions section. Dehaene (2014, pp. 8, 9) defines consciousness in terms of “conscious access”: the content of mental state/process is consciously accessible if and only if it enters awareness and becomes reportable to others. This definition consists of two notions, *awareness* and *reportability*. The property of being reportable serves to provide an informative definition of consciousness, since we can set out an objective procedure to determine whether a piece of information is reportable for its possessor. In contrast, it is unclear how “awareness” is different from “consciousness” in our ordinary conceptual understandings. Furthermore, it is unclear what behavioral standard can be used to determine whether one is aware of a piece of information, *as being different from the one for reportability*. Nevertheless, Dehaene does not seem to provide an analytic explanation of the notion of awareness. Instead, he presents a few examples of *being aware of something*. For instance, he presents an example of visual illusion and states:

Twelve dots, printed in light gray, surround a black cross. Now stare intently at the central cross. After a few seconds, you should see some of the gray dots fade in and out of existence. For a few seconds, they vanish from your awareness; then they pop back in. Sometimes the entire set goes away, temporarily leaving you with a blank page—only to return a few seconds later with a seemingly darker shade of gray. (Dehaene, 2014, p. 17)

This suggests that Dehaene leads his readers to grasp the sense of “awareness” through the examples presented in his book. If this is correct, his definition of consciousness is not entirely operational, for it does not reduce the sense of “consciousness” to reportability alone. In defining consciousness, Dehaene seems to take the example-based and essence-based approaches in combination; the former corresponds to the “awareness” part, and the latter corresponds to the “reportability” part.

Dehaene (2014, chap. 4) takes the correlation approach to the mind–body question, presenting many relevant empirical findings^11^. Based on them, he identifies four physiological markers that index whether a stimulus is consciously accessible:

First, a conscious stimulus causes an intense neuronal activation that leads to a sudden ignition of parietal and prefrontal circuits. Second, in the EEG, conscious access is accompanied by a slow wave called the P3 wave, which emerges as late as one-third of a second after the stimulus. Third, conscious ignition also triggers a late and sudden burst of high-frequency oscillations. Finally, many regions exchange bidirectional and synchronized messages over long distances in the cortex, thus forming a global brain web. (Dehaene, 2014, pp. 158, 159)

Dehaene then provides a functionalist account as to why consciousness is correlated with those physiological makers.

The human brain has developed efficient long-distance networks, particularly in the prefrontal cortex, to select relevant information and disseminate it throughout the brain. Consciousness is an evolved device that allows us to attend to a piece of information and keep it active within this broadcasting system. Once the information is conscious, it can be flexibly routed to other areas according to our current goals. Thus we can name it, evaluate it, memorize it, or use it to plan the future (Dehaene, 2014, p. 161).

This functionalist account describes how a piece of information is cognitively processed in our brain when it is consciously accessible and thereby explains why the above physiological makers occur in functional terms. This account is thus an empirically supported correlation-based answer to the mind–body question. This is, I think, the core thesis of GWT. However, Dehaene (2014, p. 161) goes beyond the empirically supported claim regarding correlation, claiming that “consciousness is brain-wide information sharing.” If we interpreted this statement literally, it would mean the identity between consciousness and the brain-wide information sharing. However, this identity claim does not directly follow from the empirically supported claim about correlation. If Dehaene (2014) defined consciousness only in terms of reportability, then the identity claim would be derived from the fact that reportability can be reductively explained in terms of brain-wide information sharing. However, Dehaene (2014) includes “awareness” in his definition of consciousness, which is supposed to be grasped through examples. It is unclear whether *the property of being aware of something* is considered to be reductively explained in functional terms, unlike reportability. Thus, Dehaene is required to explain why *the property of being aware of something* should be counted as standing in the identity relation, rather than other metaphysical relations, to the brain-wide information sharing. As in the case of IIT, we cannot properly evaluate IIT’s identity claim unless some explanation is provided.

Global workspace theory has implications for (i) the cognitive value question, (ii) the epistemological question about the consciousness of others, and (iii) the distribution question. Given that a piece of information can be flexibly routed to many brain areas only when it is consciously accessible, it is plausible to think that (i) consciousness enables its possessor to process information in such flexible manners (Dehaene, 2014, chap. 3) and that (ii) we can know about the content of the consciousness of others by detecting the information widely shared in his/her brain. (iii) It follows from GWT’s identity claim that every creature who has “brain-wide information sharing” is conscious (Dehaene, 2014, chap. 6.7).

We can clarify the relation between IIT and GWT by comparing their answers to each fundamental question. Let us take three questions, for example, the definitional question, the mind–body question, and the distribution question. For the definitional question, IIT states that consciousness is defined in terms of the five phenomenological axioms, which are supposed to capture the essential properties of consciousness. In contrast, GWT defines consciousness in terms of awareness and reportability. By comparing the two definitions of consciousness, we can examine *whether IIT and GWT have the same research subject in the first place*. For the mind–body question, IIT states that conscious experience is identical to an integrated informational structure of physical systems that instantiates the five postulates. In contrast, GWT states that consciousness is brain-wide information sharing. By comparing the two identity claims, we can examine *whether they are compatible or conflicting*. For the distribution question, IIT states that every physical system that realizes the five postulates possesses consciousness. In contrast, GWT implies that every creature who has brain-wide information sharing has consciousness. By examining whether each kind of creature overlaps, we can see *whether IIT and GWT substantially differ in what existing creatures/entities have consciousness*. In this way, we can conduct a *multidimensional comparison* between IIT and GWT. This enables us to assess the two theories systematically and comparatively in the multidimensional evaluative space.

I close this article by presenting three ideas on how to proceed with consciousness research with the help of the lists of questions and approaches proposed in this article. First, we should examine how existing theories of consciousness answer each fundamental question about consciousness and what approach their advocates adopt. By doing so, we can obtain systematic understandings of each theory of consciousness, which enable us to see what part of each theory of consciousness needs to be justified and developed. Second, we should conduct a multidimensional comparison of the existing theories of consciousness. This enables us to obtain a detailed and well-organized review of how they are related to each other. These two points have been demonstrated in the discussions of IIT and GWT. Third, each research group should clarify what question and approach to take in investigating consciousness. By doing so, they can be aware of the scope, limitation, and potential implications of their research project and also of its relations to existing theories of consciousness.

Although I believe that the proposed lists of questions and approaches contribute to the development of consciousness studies, I do not think that they are entirely satisfactory. The map of consciousness studies presented in this article can be revised and further enriched. I hope that this article also works as a springboard for a further second-order investigation on consciousness studies as being distinct from the first-order investigation on consciousness.

## Data Availability Statement

The original contributions presented in the study are included in the article/supplementary material.

## Author Contributions

The author confirms being the sole contributor of this work and has approved it for publication.

## Conflict of Interest

The authors declare that the research was conducted in the absence of any commercial or financial relationships that could be construed as a potential conflict of interest.

## Footnotes

1 Note that I do not claim that the questions belonging to distinct fundamental categories are independent of each other. Rather, they are interrelated in such a way that the answer to one question affects the scope of possible answers to other kinds of questions.

2 For the overview of philosophical issues about the content of consciousness, see Macpherson (2011) and Siegel (2016).

3 One might wonder why I do not formulate the ontological question as “what is consciousness?” The reason is that it is ambiguous in that it can also be interpreted as the definitional question and as the phenomenological question asking the essential phenomenological features of consciousness. This ambiguity of the question of “what is consciousness?” may cause confusions in consciousness studies.

4 Although I owe this division partly to Kriegel (2019), there are two differences. First, I do not introduce the distinction between intrinsic and instrumental values for the sake of simplicity. Second, I add cognitive value because (1) it does not seem to be reducible to other kinds of value and (2) the cognitive value question has actually been discussed in scientific consciousness studies (Kanai et al., 2019).

5 Husserlian Phenomenology can be counted as introspective on this broad definition of introspection (Breyer and Gutland, 2016, p. 13; Gutland, 2018).

6 It is controversial whether some kind of inference is always required to know what others consciously experience. This paper sets aside this issue for simplicity. See McDowell (1983) for an argument against the necessity of inference.

7 This is not the original form of Pitt’s argument but its possible reformulation.

8 One might wonder why the question of “how it is possible for us to know about consciousness,” which has been much discus-sed in philosophy, is not included in the list of subcategories of the epistemological question. The reason is that the “how possible” question should be interpreted as a form of the ontological question, namely: what relation must hold between consciousness and the world (including our body and brain) given that it is possible for us to know about consciousness?

9 For more details of axioms in IIT, see Oizumi et al. (2014); Tononi et al. (2016), and Miyahara and Witkowski (2019).

10 For the details of the postulates, see Oizumi et al. (2014).

11 It is necessary to address the content question before exploring the correlation between the contents of consciousness and neural/informational features of brains. Dehaene (2014, p. 41–45) takes the observation approach to address the content question, emphasizing the availability of subjective reports from experimental participants as objective data.
